# Integrating Deficit Irrigation and Bacterial Inoculation to Mitigate Water Stress and Enhance Maize Productivity in Semiarid Regions

**DOI:** 10.3390/plants15091309

**Published:** 2026-04-24

**Authors:** Danilo B. Nogueira, José Lucas P. da Silva, Aelton B. Giroldo, Ênio F. França e Silva, Gerônimo F. da Silva, Geocleber G. de Sousa, Rafaela da S. Arruda, Kleyton C. de Sousa, Fernando F. Putti, Alexsandro O. da Silva

**Affiliations:** 1 Departamento de Engenharia Agrícola, Universidade Federal do Ceará, Fortaleza 60455-750, Brazil; 2 Departamento de Engenharia Agrícola, Universidade Federal Rural de Pernambuco, Recife 52171-960, Brazil; jose.lucaspereira@ufrpe.br (J.L.P.d.S.); alexsandro@ufc.br (A.O.d.S.); 3 Empresa Brasileira de Pesquisa Agropecuária (Embrapa), São Luís 65020-500, Brazil; aelton.giroldo@embrapa.br; 4 Instituto de Desenvolvimento Rural, Universidade da Integração Internacional Lusofonia Afro-Brasileira, Redenção 62790-000, Brazil; 5 Fazenda Experimental Vale do Curu, Universidade Federal do Ceará, Pentecoste 62640-000, Brazil; 6 Faculdade de Ciências e Engenharias, Universidade Estadual Paulista “Júlio de Mesquita Filho”, Campus de Tupã, Tupã 17602-496, Brazil

**Keywords:** water deficit, *Zea mays* L., *Bacillus aryabhattai*, *Azospirillum brasilense*, yield

## Abstract

Water scarcity is one of the main constraints on maize production in semiarid regions, making it essential to adopt management strategies that reconcile water savings, crop resilience, and economic viability. This study evaluated the effects of deficit irrigation strategies integrated with the use of bioinputs on physiological, productive, and economic parameters of maize grown under field conditions in the Brazilian semiarid region over two growing seasons (2023 and 2024). The experiment was conducted using a randomized complete block design with a split-plot arrangement. Irrigation strategies comprised full irrigation (FI; 100% of crop water requirements), continuous deficit irrigation (RD50%; 50% throughout the crop cycle), and stage-specific controlled deficit irrigation (50%) imposed during the vegetative (CDV50%), flowering/grain formation (CDF50%), and grain-filling (CDG50%) stages, while seed treatments involved inoculation with *Bacillus aryabhattai*, coinoculation with *B*. *aryabhattai* + *Azospirillum brasilense*, and control treatments. Physiological variables, yield components, water use efficiency, the crop sensitivity coefficient to water deficit (Ky), and economic indicators were assessed. Controlled deficits irrigation, particularly under CDV50%, maintained grain yield comparable to FI (6465.80 kg ha^−1^, in second growing season), whereas RD50% reduced yield in 26%. Inoculation treatments enhanced gas exchange, carboxylation efficiency, and water use efficiency, resulting in higher agricultural income under specific production systems. The CDV50% strategy combined with coinoculation showed the greatest potential as a sustainable approach for maize production in semiarid environments and reduced the water use by up to 18.9%.

## 1. Introduction

Maize (*Zea mays* L.) is one of the most important crops worldwide, standing out both for its strategic role in global food security and for its broad use in animal nutrition and as an industrial raw material [1,2]. In Brazil, this cereal has high socioeconomic relevance, with an estimated production of 115.7 million tons in the 2023/2024 growing season and an average yield of approximately 5887 kg ha^−1^ [3]. In the Brazilian Northeast in particular, maize plays a central role in family-based agriculture, contributing to local food supply and income generation [3].

Maize is highly sensitive to water deficit, which is considered one of the main abiotic factors limiting crop productivity, especially in arid and semiarid regions [4,5,6]. Water stress occurs when crop water demand exceeds soil water availability, leading to cellular dehydration, stomatal closure, inhibition of photosynthesis, and reduced leaf expansion [7]. These negative effects become more pronounced at critical phenological stages, such as flowering and grain filling [8,9]. This scenario is further exacerbated by climate change, which increases rainfall irregularity and intensifies the challenges to the sustainability of agricultural production in semiarid environments.

In this context, water management strategies such as deficit irrigation have been widely investigated because they combine water savings with only minor reductions in crop productivity [10,11]. This technique involves applying irrigation depths below the full crop water requirements in a controlled and planned manner, exploiting the greater tolerance of the plant at specific stages of its development. However, the impact of deficit irrigation on maize cultivated in semiarid regions still lacks integrated studies that simultaneously address physiological, agronomic, and socioeconomic aspects.

In parallel, the use of bioinputs, particularly Plant Growth-Promoting Bacteria (PGPB), has emerged as a sustainable alternative to enhance plant resilience under stress conditions [12,13]. Species such as *B. aryabhattai* and *A. brasilense* are noteworthy, acting through multiple mechanisms, including biological nitrogen fixation, nutrient solubilization, phytohormone production, and modulation of plant responses to stress. *A. brasilense* is widely recognized for its ability to fix atmospheric nitrogen and stimulate root development, while *B. aryabhattai* has been associated with nutrient mobilization, including phosphorus and potassium solubilization, as well as the production of metabolites that increase plant tolerance to abiotic stress [14,15,16].

Moreover, the coinoculation of these bacteria may generate synergistic effects, amplifying gains in plant growth and water use efficiency [17]. Nevertheless, although promising, the interactions between bioinputs and different levels of water deficit are not yet fully elucidated, with divergent results reported regarding the magnitude of their physiological and productive effects [18,19].

Given these knowledge gaps, this study was conducted under the hypothesis that inoculation and coinoculation with *B. aryabhattai* and *A. brasilense* could mitigate the effects of water deficit in maize by improving physiological and productive parameters and increasing water use efficiency. Additionally, we investigated whether the integration of bioinputs with deficit irrigation strategies could reduce the crop sensitivity coefficient to water deficit (Ky) and provide economic gains in production systems typical of semiarid regions. Therefore, this study aimed to evaluate the combined effects of deficit irrigation strategies and bacterial inoculation (*B. aryabhattai* and *A*. *brasilense*), applied individually and in coinoculation, on maize performance under semiarid conditions. Specifically, the study assessed agronomic, physiological, water use efficiency, and economic responses in order to identify strategies capable of mitigating water stress and improving crop productivity.

## 2. Results

### 2.1. Agronomic Components and Grain Yield

Analysis of variance indicated that, in the first growing season (2023), a significant effect was observed only for the irrigation factor on grain yield per plant (GYP) and grain yield (YIELD), as well as for the irrigation × bioinputs interaction on fresh ear mass without husk (MFS) (Table 1). In the second growing season (2024), the irrigation factor significantly affected ear diameter (ED), number of kernels per row (NKR), fresh ear mass with husk (MFC) and fresh ear mass without husk (MFS), both determined immediately after harvest, grain yield per plant (GYP), and grain yield (YIELD). No significant effects of bioinputs or of the irrigation × bioinputs interaction were observed for the remaining variables (Table 1).

Overall, these results indicate that irrigation was the primary factor driving variability in agronomic traits, with a more pronounced effect in the second growing season. In contrast, the limited significance of bioinputs suggests a more variable or context-dependent influence on yield-related parameters under the evaluated conditions.

**Figure 1:**
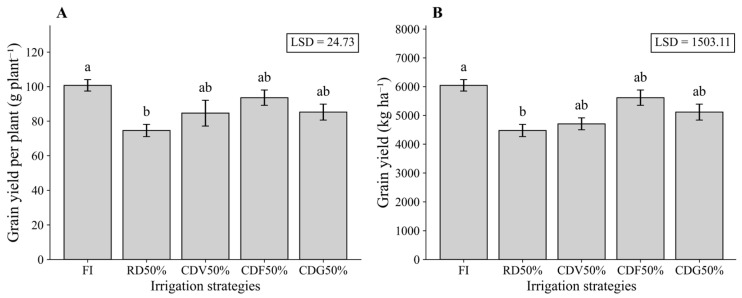
Grain yield per plant (**A**) and grain yield (**B**) of maize under different irrigation strategies during the first growing season (2023). Means followed by the same letter do not differ statistically according to Tukey’s test (*p* ≤ 0.05). LSD: Least Significant Difference; FI—Full irrigation throughout the entire crop cycle; RD50%—Regular deficit irrigation throughout the entire crop cycle; CDV50%—Controlled deficit irrigation during the vegetative growth stage; CDF50%—Controlled deficit irrigation during the flowering and grain formation stage; CDG50%—Controlled deficit irrigation during the grain-filling stage.

In the first growing season, GYP was reduced under RD50% (74.54 g plant^−1^) compared with FI (100.73 g plant^−1^), whereas irrigation strategies with controlled water deficit by phenological stage, CDV50%, CDF50%, and CDG50%, showed mean values statistically similar to FI (84.62, 93.58, and 85.23 g plant^−1^, respectively) (Figure 1A). For YIELD, FI reached 6043.72 kg ha^−1^, which was statistically similar to CDV50% (4705.78 kg ha^−1^), CDF50% (5615.00 kg ha^−1^), and CDG50% (5114.12 kg ha^−1^). In contrast, RD50% reduced YIELD to 4472.68 kg ha^−1^ (−26% relative to FI) (Figure 1B). These results indicate that stage-specific deficit irrigation mitigated yield losses, whereas continuous water restriction negatively affected maize productivity.

As no significant effects of bioinputs or irrigation × bioinputs interaction were observed for these variables, the results are presented considering only the irrigation factor. This approach was adopted to facilitate data visualization and focus on the main sources of variation affecting that significantly these traits.

**Figure 2:**
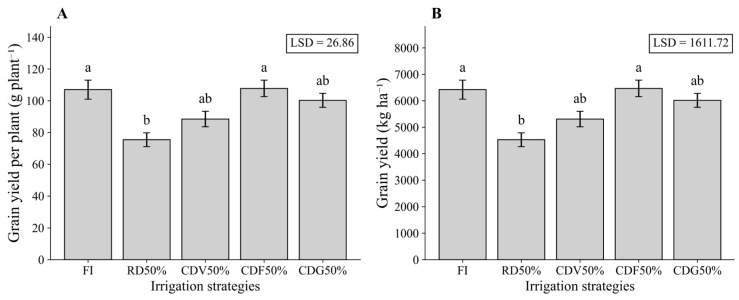
Grain yield per plant (**A**) and grain yield (**B**) of maize under different irrigation strategies during the second growing season (2024). Means followed by the same letter do not differ statistically according to Tukey’s test (*p* ≤ 0.05). LSD: Least Significant Difference; FI—Full irrigation throughout the entire crop cycle; RD50%—Regular deficit irrigation throughout the entire crop cycle; CDV50%—Controlled deficit irrigation during the vegetative growth stage; CDF50%—Controlled deficit irrigation during the flowering and grain formation stage; CDG50%—Controlled deficit irrigation during the grain-filling stage.

In the second growing season, GYP and YIELD also responded significantly to irrigation strategies (Figure 2). The CDV50% treatment presented the highest mean values (GYP = 107.76 g plant^−1^; YIELD = 6465.80 kg ha^−1^), whereas RD50% resulted in the lowest productivity, differing significantly from FI (Figure 2B). These results reinforce the advantage of applying water deficit during the vegetative stage, while deficits imposed during grain filling had a more detrimental effect on maize yield.

The irrigation depths are detailed in the Section 4, but it is important to highlight that deficit irrigation strategies resulted in substantial reductions in total water applied compared with full irrigation (FI). In 2023, full irrigation (FI) corresponded to 2629.42 m^3^ ha^−1^, whereas the CDF50% treatment used 2373.56 m^3^ ha^−1^, representing a reduction of approximately 9.7% in total water volume. In 2024, FI reached 2282.12 m^3^ ha^−1^, while CDF50% required 2009.82 m^3^ ha^−1^, corresponding to a reduction of approximately 11.9%.

These differences highlight the potential of deficit irrigation strategies at specific phenological stages to reduce water consumption in semi-arid conditions without significantly reducing production levels.

**Figure 3:**
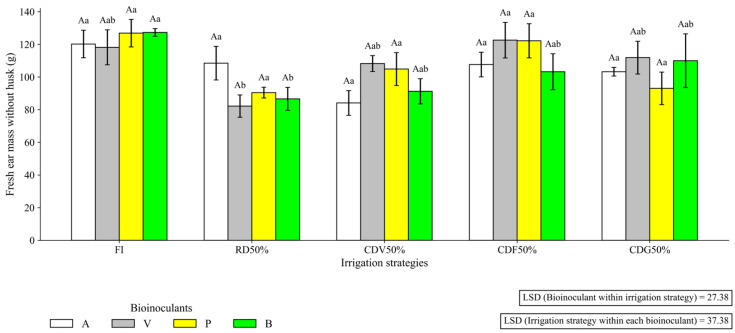
Fresh ear mass without husk (MFS) of maize under different irrigation strategies and Plant Growth-Promoting Bacteria (PGPB) bioinputs during the first growing season (2023). Uppercase letters compare means of plants receiving different bioinputs within the same irrigation strategy. Lowercase letters compare mean values of plants grown under different irrigation strategies within each bioinput treatment. LSD: Least Significant Difference; FI—Full irrigation throughout the entire crop cycle; RD50%—Regular deficit irrigation throughout the entire crop cycle; CDV50%—Controlled deficit irrigation during the vegetative growth stage; CDF50%—Controlled deficit irrigation during the flowering and grain formation stage; CDG50%—Controlled deficit irrigation during the grain-filling stage. A—Inoculation with *B. aryabhattai*; V—Coinoculation with *B. aryabhattai* + *A. brasilense*; P—Positive control, with mineral nitrogen fertilization (urea, 45% N, applied at the recommended rate based on soil analysis), without bioinputs; B—Negative control, without mineral nitrogen fertilization or inoculation.

In the first growing season, the interaction between irrigation and bioinputs for MFS is presented in Figure 3. In general, inoculation treatments tended to mitigate the reduction in MFS under water deficit conditions, although responses varied depending on the irrigation strategy.

**Figure 4:**
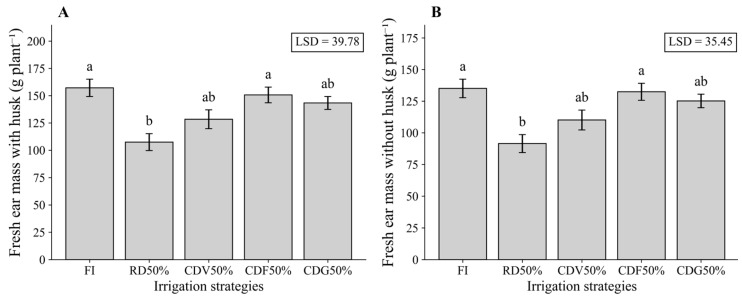
Fresh ear mass with husk (**A**) and without husk (**B**) of maize under different irrigation strategies during the second growing season (2024). Means followed by the same letter do not differ statistically according to Tukey’s test (*p* ≤ 0.05). LSD: Least Significant Difference; FI—Full irrigation throughout the entire crop cycle; RD50%—Regular deficit throughout the entire crop cycle; CDV50%—Controlled deficit during the vegetative growth stage; CDF50%—Controlled deficit during the flowering and grain formation stage; CDG50%—Controlled deficit during the grain-filling stage.

For MFC and MFS in the second growing season, FI showed the highest mean values, whereas RD50% significantly reduced both variables. The CDV50%, CDF50%, and CDG50% treatments maintained intermediate values, exhibiting similar response patterns for MFC and MFS (Figure 4A,B). These results indicate that controlled deficit irrigation partially alleviated the negative effects of water restriction on ear biomass.

**Figure 5:**
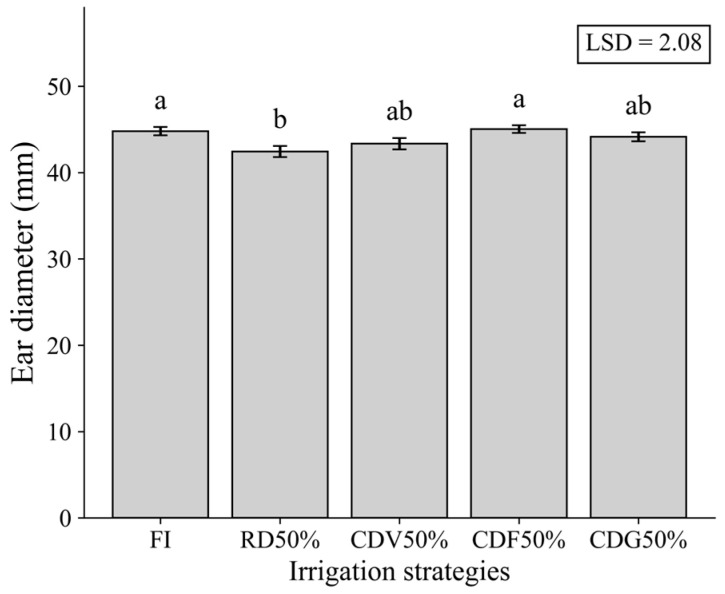
Ear diameter (ED) of maize under different irrigation strategies during the second growing season (2024). Means followed by the same letter do not differ statistically according to Tukey’s test (*p* ≤ 0.05). LSD: Least Significant Difference; FI—Full irrigation throughout the entire crop cycle; RD50%—Regular deficit throughout the entire crop cycle; CDV50%—Controlled deficit during the vegetative growth stage; CDF50%—Controlled deficit during the flowering and grain formation stage; CDG50%—Controlled deficit during the grain-filling stage.

**Figure 6:**
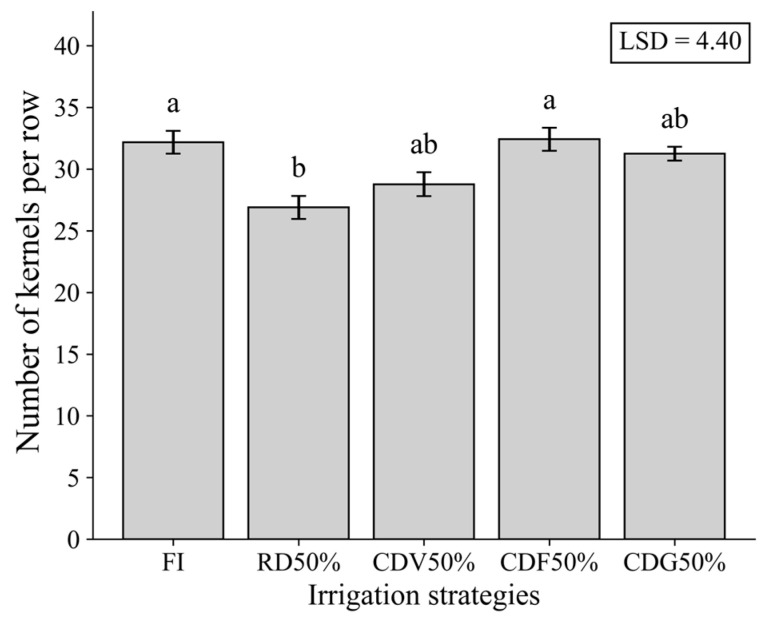
Number of kernels per row (NKR) in maize under different irrigation strategies during the second growing season (2024). Means followed by the same letter do not differ statistically according to Tukey’s test (*p* ≤ 0.05). LSD: Least Significant Difference; FI—Full irrigation throughout the entire crop cycle; RD50%—Regular deficit throughout the entire crop cycle; CDV50%—Controlled deficit during the vegetative growth stage; CDF50%—Controlled deficit during the flowering and grain formation stage; CDG50%—Controlled deficit during the grain-filling stage.

For ear diameter (ED) and number of kernels per row (NKR) in the second growing season, irrigation strategies significantly affected both variables. FI resulted in the highest mean values, while RD50% reduced ED and NKR, although values were statistically similar to those observed under CDV50% and CDG50% (Figure 5 and Figure 6).

These results suggest that water deficit, particularly when applied continuously, negatively influences key yield components, whereas controlled deficit strategies tend to mitigate these effects.

### 2.2. Irrigation Water Use Efficiency (IWP)

**Figure 7:**
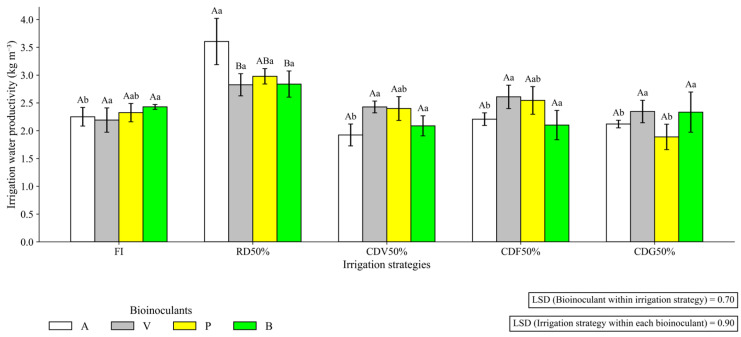
Irrigation water productivity (IWP) of maize under different irrigation strategies and Plant Growth-Promoting Bacteria (PGPB) bioinputs during the first growing season (2023). Uppercase letters compare means of plants receiving different bioinputs within the same irrigation strategy. Lowercase letters compare mean values of plants grown under different irrigation strategies within each bioinput treatment. LSD: Least Significant Difference; FI—Full irrigation throughout the entire crop cycle; RD50%—Regular deficit throughout the entire crop cycle; CDV50%—Controlled deficit during the vegetative growth stage; CDF50%—Controlled deficit during the flowering and grain formation stage; CDG50%—Controlled deficit during the grain-filling stage. A—Inoculation with *B. aryabhattai*; V—Coinoculation with *B. aryabhattai* + *A. brasilense*; P—Positive control, with mineral Nitrogen fertilization (urea, 45% N, applied at the recommended rate based on soil analysis), without bioinputs; B—Negative control, without mineral N fertilization or inoculation.

In the first growing season, there was a significant interaction between irrigation and bioinputs for IWP (*p* ≤ 0.01). Under RD50%, inoculation with *B. aryabhattai* (A) increased IWP to 3.61 kg m^−3^, outperforming the control without bioinputs and without mineral N (B: 2.84 kg m^−3^) (Figure 7).

### 2.3. Physiological Indices (SPAD and Gas Exchange)

No significant effects of the studied factors were detected for the SPAD chlorophyll index in any of the evaluations.

**Figure 8:**
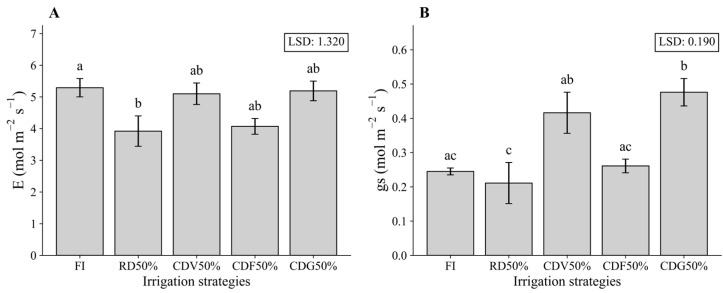
Transpiration rate (E, (**A**)) and stomatal conductance (gs, (**B**)) of maize under different irrigation strategies at 64 DAS during the first growing season (2023). Means followed by the same letter do not differ statistically according to Tukey’s test (*p* ≤ 0.05). FI—Full irrigation throughout the entire crop cycle; RD50%—Regular deficit throughout the entire crop cycle; CDV50%—Controlled deficit during the vegetative stage; CDF50%—Controlled deficit during the flowering and grain formation stage; CDG50%—Controlled deficit during the grain-filling stage.

Regarding gas exchange, in the first growing season (64 DAS), E and gs responded significantly to both irrigation and bioinputs, whereas Ci was affected by irrigation and by the irrigation × bioinputs interaction (Table 2). The highest rate for E was observed in FI, however statistically equal to the averages observed in the other treatments with deficits in specific growth phases CDV50%, CDF50% and CDG50% (Figure 8A); gs, in turn, presented the highest averages in CDV50% and CDG50% (Figure 8B).

**Figure 9:**
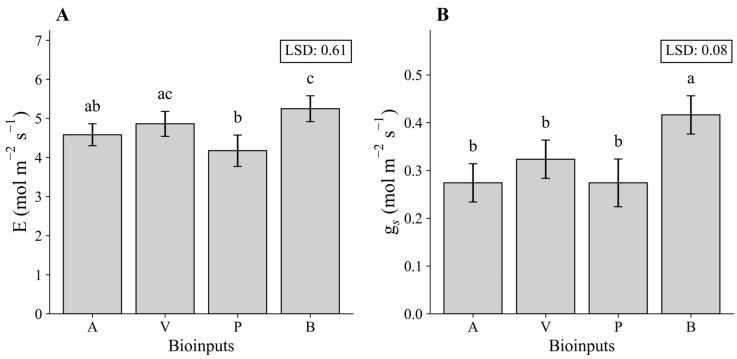
Transpiration rate (E) (**A**) and stomatal conductance (gs) (**B**) of maize under different plant growth-promoting bioinputs during the first growing season at 64 DAS (2023). Means followed by the same letter do not differ statistically according to Tukey’s test (*p* ≤ 0.05). A—Inoculation with *B. aryabhattai*; V—Coinoculation with *B. aryabhattai* + *A. brasilense*; P—Positive control, with mineral nitrogen addition (urea, 45% N, applied at the recommended rate according to soil analysis), without bioinputs; B—Negative control, without mineral N or inoculation.

Regarding the response of the variables E and gs to the tested bio-inputs, it was found that the negative control, without inoculation or nitrogen application, provided the highest mean for E, which was statistically equal to the mean observed in V (Figure 9A); Figure 9B shows a similar pattern of behavior for the variable gs in relation to the treatments with bio-inputs.

**Figure 10:**
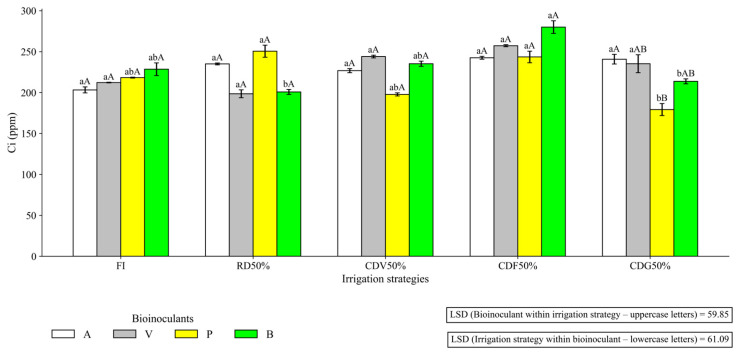
Internal CO_2_ concentration (Ci) in maize under different irrigation strategies and plant growth-promoting bioinputs during the first growing season at 64 DAS (2023). Uppercase letters compare mean values of plants subjected to different plant growth-promoting bioinputs within the same irrigation strategy. Lowercase letters compare mean values of plants grown under different irrigation strategies within each bioinput treatment. FI—Full irrigation throughout the entire crop cycle; RD50%—Regular deficit throughout the entire crop cycle; CDV50%—Controlled deficit during the vegetative stage; CDF50%—Controlled deficit during the flowering and grain formation stage; CDG50%—Controlled deficit during the grain-filling stage. A—Inoculation with *B. aryabhattai*; V—Coinoculation with *B. aryabhattai* + *A. brasilense*; P—Positive control, with mineral nitrogen addition (urea, 45% N, applied at the recommended rate according to soil analysis), without bioinputs; B—Negative control, without mineral N or inoculation.

Still in the first cycle, Figure 10 shows that, despite the occurrence of higher averages associated with the negative control, treatments A and V provided increases in Ci under CDV50% and CDG50%.

**Figure 11:**
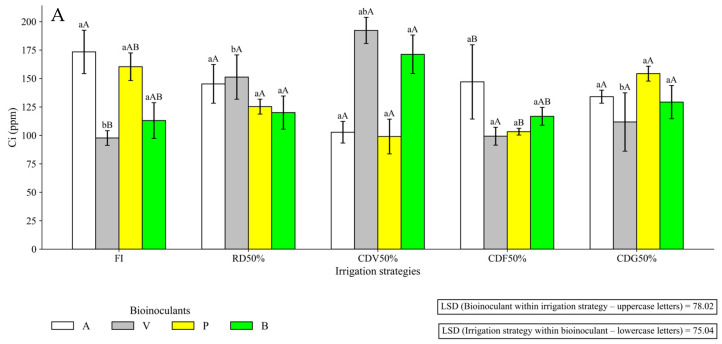

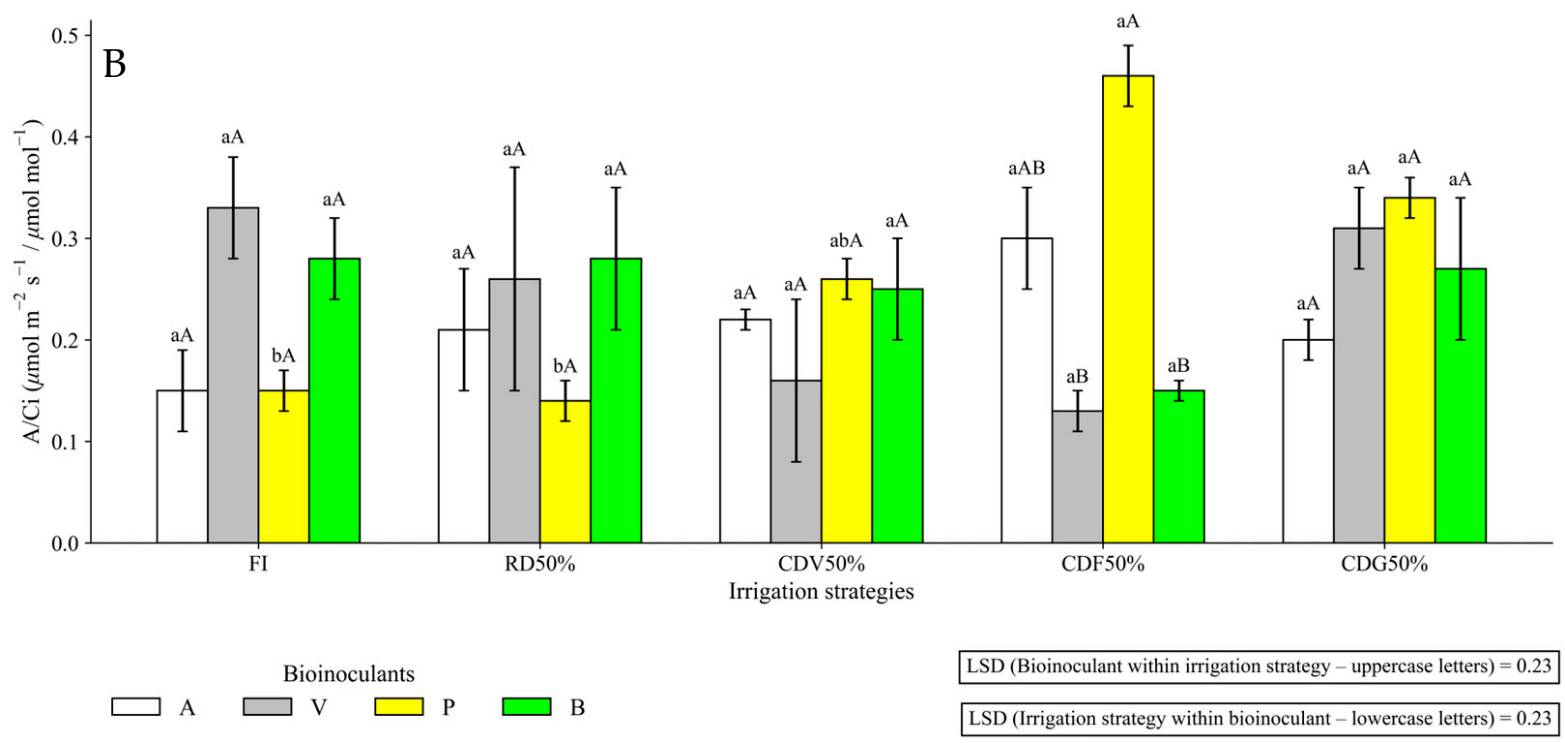
Internal CO_2_ concentration (Ci) (**A**) and the ratio between photosynthesis and internal CO_2_ concentration (A/Ci) (**B**) in maize under different irrigation strategies and plant growth-promoting bioinputs during the second growing season at 72 DAS (2024). Uppercase letters compare mean values of plants subjected to different plant growth-promoting bioinputs within the same irrigation strategy. Lowercase letters compare mean values of plants grown under different irrigation strategies within each bioinput treatment. FI—Full irrigation throughout the entire crop cycle; RD50%—Regular deficit throughout the entire crop cycle; CDV50%—Controlled deficit during the vegetative stage; CDF50%—Controlled deficit during the flowering and grain formation stage; CDG50%—Controlled deficit during the grain-filling stage. A—Inoculation with *B. aryabhattai*; V—Coinoculation with *B. aryabhattai* + *A. brasilense*; P—Positive control, with mineral nitrogen addition (urea, 45% N, applied at the recommended rate according to soil analysis), without bioinputs; B—Negative control, without mineral N or inoculation.

In the second growing season (72 DAS), only Ci and A/Ci showed a significant interaction effect; A, E, and gs were not significantly affected (Table 2; Figure 11).

### 2.4. Crop Sensitivity Coefficient to Water Deficit (Ky)

Ky values varied across growing seasons, phenological stages, and bioinputs (Table 3). In the first growing season (2023), high Ky values were observed under CDG50%-P (Ky = 2.61) and CDF50%-B (Ky = 2.25), contrasting with low to moderate values under RD50% (Ky = 0.25–0.79) and CDV50%-V (Ky = 0.54). In the second growing season (2024), high sensitivity was observed under CDG50% (Ky = 3.03–4.33), whereas combinations such as RD50%-P (Ky = 0.05) and CDV50%-P (Ky = 0.15) exhibited low sensitivity (Table 3). Cases with negative Ky values were excluded according to the methodological note.

### 2.5. Economic Results

Gross production value (GPV) and the parameters used to calculate farmer income (FIc) for each production system are presented in Table 4 (2023) and Table 5 (2024). In 2023, the CDV50%-V treatment resulted in the highest FIc among the evaluated treatments; in 2024, CDV50%-V again stood out, with FIc = R$13,764.92 ha^−1^, outperforming all other treatments. The linear equations relating FIc to cultivated area are presented in Table 6.

The minimum area required to ensure social reproduction (MA-SR) varied widely among treatments (Table 7). In 2023, the lowest MA-SR values were observed for CDF50%-A (4.2 ha), CDV50%-A (4.7 ha), and FI-A (4.8 ha), whereas markedly high values occurred under CDG50% (e.g., 104.9 ha in CDG50%-A). In 2024, the lowest MA-SR values included CDF50%-A (4.0 ha), CDV50%-V (4.4 ha), and FI-A (7.0 ha), while the highest MA-SR values were again recorded under CDG50% (31.8–73.1 ha) (Table 7).

## 3. Discussion

Maize grain yield was significantly affected by irrigation strategies, confirming the high sensitivity of the crop to continuous water deficit. The reduction in GYP and YIELD under RD50% in both growing seasons indicates that uniform water restriction compromises vegetative and reproductive growth, corroborating reports by [20] that regular water deficit cumulatively impairs physiological functions, resulting in yield losses of up to 40% in semiarid environments. In the present study, the yield loss observed relative to full irrigation (FI) in the first growing season (approximately—26%) is consistent with the findings of [12] who reported that water deficit reduces soil water potential and, consequently, limits soil solution availability. This constraint restricts nutrient uptake and physiological processes, thereby markedly affecting crop productivity.

In contrast, the adoption of controlled deficit strategies (CDV50%, CDF50%, and CDG50%) revealed greater adaptive flexibility of maize, with particular emphasis on CDV50%, which resulted in yields similar to or even higher than those obtained under FI, depending on the growing season. These results are supported by [21], who highlighted that limited water availability during the pre-flowering period may affect vegetative structure development but does not necessarily reduce final yield when water deficit is properly controlled. In that study, the authors concluded that, under certain conditions, the yield of specific hybrids can be maintained even under moderate water stress, emphasizing the importance of selecting adapted genotypes. Similarly, [22] reported that maize productivity in the Brazilian semiarid region, where water stress is recurrent, can be sustained through efficient management practices. Although climate variability and irregular rainfall represent major challenges, the adoption of appropriate agronomic practices, such as the selection of drought-tolerant cultivars and efficient irrigation strategies, can result in yields comparable to those obtained under full irrigation [5]. Thus, the findings of the present study are consistent with the existing literature, indicating that controlled water deficit imposed during critical stages of maize development does not necessarily compromise final grain yield.

The effects of bioinputs on agronomic variables were more specific, with a significant interaction observed only for MFS in the first growing season. This partial response may be attributed to seasonal variations in soil microbiota and environmental conditions, which influence the survival and activity of inoculated microorganisms. Nevertheless, the results are consistent with studies by [14], who reported variable yield gains under inoculation with *A. brasilense* depending on environmental conditions. Previous studies by [23,24,25], have also reported positive effects of bacterial inoculation on plant growth under low water availability, partially corroborating the findings of this study, although the responses observed here were more modest than those reported in some of these investigations.

Irrigation water productivity (IWP) was influenced by the irrigation × bioinputs interaction, particularly in the first growing season. Under regular water deficit conditions (RD50%), inoculation with *B. aryabhattai* resulted in a 21.3% increase in irrigation water productivity (3.61 kg m^−3^) compared with the negative control without bioinputs or urea application (2.84 kg m^−3^). This outcome is especially relevant for semiarid regions, where water scarcity requires strategies that maximize productivity per unit volume of applied water. Previous studies [14,15] have shown that Plant Growth-Promoting Bacteria (PGPB) enhance root hydraulic conductivity and regulate stomatal opening and closure processes, thereby improving water use efficiency.

However, coinoculation did not provide a consistent advantage over the isolated use of *B. aryabhattai*. Earlier studies emphasize that coinoculation efficiency depends on strain compatibility and adaptation to edaphoclimatic conditions, as competition for ecological niches or resources may occur [25]. This finding suggests that, in semiarid environments, the selection of microbial consortia should be based on regional field trials rather than solely on evidence from other contexts. Nevertheless, the results indicate that bioinputs constitute a strategic tool for reconciling water savings with the maintenance of productivity, although the magnitude of the benefits is modulated by crop cycle conditions and the surrounding environment.

Physiological responses further corroborate the importance of water management and bacterial inoculation in regulating gas exchange. In the first growing season, the reduction in E and gs under RD50% confirms stomatal closure as a primary defense mechanism against water deficit, reducing transpiration losses but simultaneously limiting carbon assimilation. Previous studies indicate that this adjustment, while protective in the short term, may compromise net photosynthesis and, consequently, crop productivity [26].

The positive modulation observed in inoculated plants indicates that bioinputs such as *B. aryabhattai* and *A. brasilense* can mitigate these effects by promoting greater stomatal opening under moderate water stress conditions. Studies have shown that rhizospheric microorganisms can increase the synthesis of phytohormones, such as abscisic acid and auxins, thereby directly influencing stomatal dynamics [12]. In addition, other authors have reported that this interaction stimulates phytohormone production and enhances root growth, resulting in improved plant water status and greater CO_2_ assimilation, which sustains gains in physiological efficiency [27].

In the second growing season, the most pronounced effects were observed for Ci and A/Ci, variables directly associated with the biochemical efficiency of photosynthesis. The increase in A/Ci in inoculated plants under water deficit indicates higher carboxylation efficiency, suggesting that bioinputs contributed to optimizing Rubisco activity and the processes associated with carbon assimilation. According to [28], this mechanism results from the production of bioactive compounds that modulate metabolic pathways and reduce oxidative stress, thereby favoring the maintenance of photosynthesis under adverse conditions.

These results confirm that inoculation and coinoculation do not act solely on root growth and nutrient uptake but also promote physiological adjustment at the leaf level, reinforcing the potential of bioinputs as water stress mitigators in semiarid environments.

From an economic perspective, the Ky values obtained in this study highlight the grain-filling stage as the most sensitive to water deficit, with values exceeding 3.0 under CDG50% in the second growing season, indicating disproportionate yield losses relative to reductions in evapotranspiration. This finding is consistent with [29], who classified maize as highly sensitive to water deficit during this stage, and was further corroborated by [30], who reported yield reductions of up to 50% under reproductive-stage water stress.

On the other hand, the low Ky values observed during vegetative stages confirm greater crop tolerance, in agreement with [31], who reported physiological resilience and lower yield penalties when water deficit was imposed before flowering. The influence of bioinputs is particularly relevant in this context: in some cases, lower Ky values were recorded in inoculated treatments, suggesting that the rhizospheric microbiota may reduce crop sensitivity to water deficit. This finding reinforces the hypothesis that bioinputs not only promote direct gains in plant growth but also contribute to enhancing crop resilience to stress by modulating the relationship between evapotranspiration and productivity.

The economic results confirm that the financial viability of maize production in semiarid environments is directly associated with the integration of efficient water management and the use of bioinputs. In both growing seasons, the highest FIc values were obtained under controlled deficit strategies (especially CDV50%), whereas the lowest values were associated with continuous water deficit and, in particular, with water deficit during the grain-filling stage (CDG50%).

The prominence of CDV50%-V in the second growing season, with FIc values higher than those of all other treatments, demonstrates that the combination of water savings and bioinput use can maximize economic returns. Similar results have been reported with inoculation using *A. brasilense*, which operationally increased profit and profitability indices in field studies conducted in Brazil [32].

The high MA-SR values recorded under CDG50% reinforce the negative impact of adopting this practice in family-based agricultural systems, whose viability depends on relatively small but productive land areas. This finding is consistent with evidence showing that water deficit during the reproductive stage severely compromises maize production and the economic viability of smallholder farmers in semiarid regions. Several studies report much greater yield losses when water deficit occurs during the reproductive phase [33], and applied research in the Brazilian semiarid region has demonstrated that drought shocks substantially reduce cultivated area and production value for farming households, thereby increasing their economic vulnerability [34].

Thus, the results of this study not only confirm the importance of appropriate water management strategies but also demonstrate that the use of bioinputs can represent a strategic alternative to increase profitability in water-restricted areas, provided that water deficit is applied during less critical stages of the crop cycle.

In summary, the integration of controlled deficit irrigation and bioinputs shows strong potential to enhance yield, water use efficiency, and economic sustainability in semiarid environments. However, the variability observed between growing seasons highlights the influence of meteorological conditions and the need for long-term, multi-environment studies. In addition, the efficiency of coinoculation appears to depend on microbial interactions and environmental factors, requiring further investigation into strain compatibility. Future research should also incorporate environmental indicators and evaluate different genetic backgrounds to optimize bioinput–water management strategies. These findings reinforce the importance of advancing integrated approaches to improve maize resilience and support sustainable agricultural systems in semiarid regions.

## 4. Materials and Methods

### 4.1. Study Area

**Figure 12:**
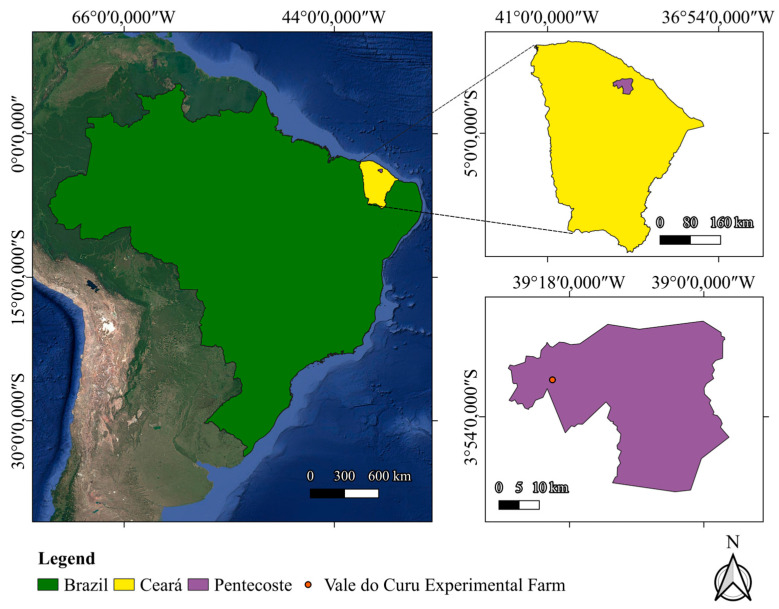
Location of the study area at the Vale do Curu Experimental Farm, Pentecoste, Ceará, Brazil.

The study was conducted under field conditions at the Vale do Curu Experimental Farm (FEVC), affiliated with the Center for Agricultural Sciences of the Federal University of Ceará, located in the municipality of Pentecoste, Ceará State, Brazil (03°49′08″ S; 39°20′02″ W). The experimental trial was carried out over two growing seasons, from August to November 2023 and from September to December 2024. Figure 12 shows the location of the experimental area and an aerial view of the field experiment.

**Figure 13:**
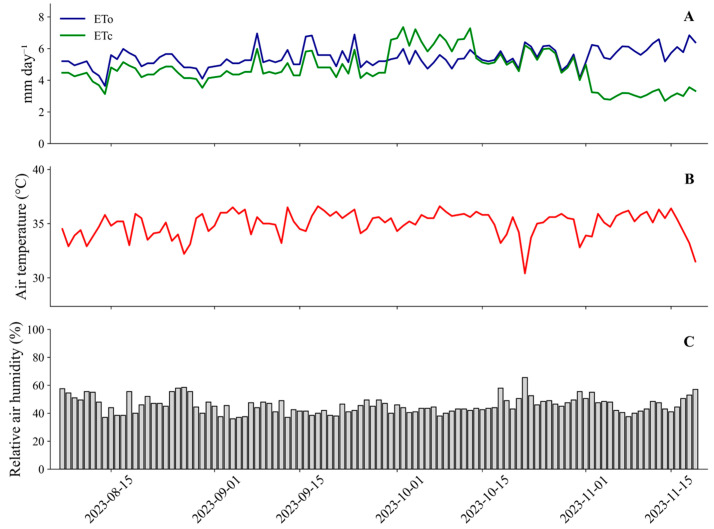
Reference evapotranspiration (ETo) and crop evapotranspiration (ETc) (**A**), air temperature (**B**), and relative humidity (**C**) during the first growing season of the experiment (7 August–19 November 2023).

**Figure 14:**
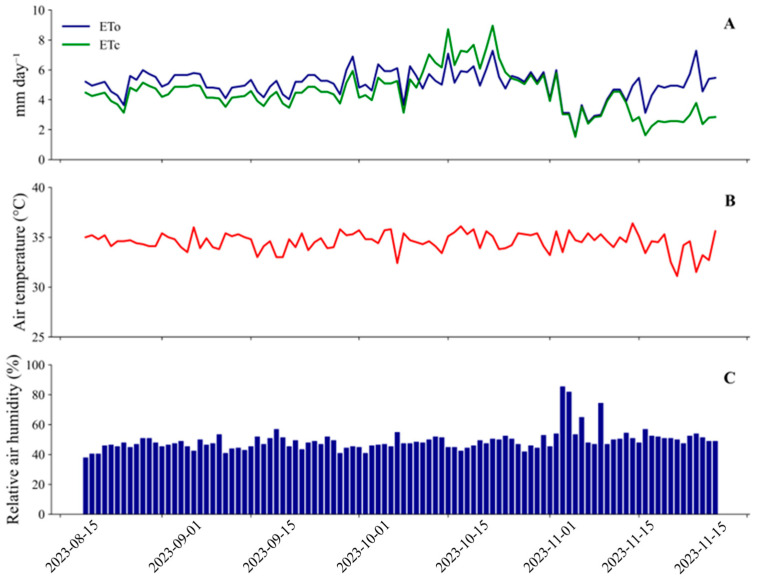
Reference evapotranspiration (ETo) and crop evapotranspiration (ETc) (**A**), air temperature (**B**), and relative humidity (**C**) during the second growing season of the experiment (19 September–27 December 2024).

The regional climate is classified as BSw’h’ (hot semiarid) according to the Köppen classification. During the two growing seasons (August to November 2023 and September to December 2024), typical seasonal rainfall patterns were recorded, with no rainfall during the first cycle and the occurrence of a single isolated rainfall event of 32 mm during the second cycle. Mean air temperature, relative humidity, and reference evapotranspiration remained within the typical range for the region, reflecting regional climatic variability and providing support for irrigation management. Meteorological data recorded during the first and second growing seasons are presented in Figure 13 and Figure 14, respectively.

The soil of the experimental area was classified as a Fluvent (Neossolo Flúvico) with a loamy texture [35], flat relief, and neutral pH, which is suitable for maize cultivation. The physical and chemical soil characterization of the 0–20 cm layer is presented in Table 8.

Soil samples were collected as a composite sample from the experimental area for initial characterization, and therefore the results represent single analytical values.

### 4.2. Experimental Design and Treatments

The experiment was conducted using a randomized complete block design in a split-plot arrangement, with four replications.

The plots corresponded to five irrigation strategies:

- Full irrigation (FI), supplying 100% of crop water requirements throughout the entire crop cycle;
- Regular deficit (RD50%), supplying 50% of crop water requirements throughout the entire crop cycle;
- Controlled deficit during the vegetative stage (CDV50%);
- Controlled deficit during the flowering and grain formation stage (CDF50%);
- Controlled deficit during the grain-filling stage (CDG50%).

In treatments with different irrigation strategies, water deficit (50% of crop water requirements) was imposed only during the designated phenological stage, while full irrigation (100%) was applied during the remaining growth stages.

The subplots received two input management strategies applied via seed treatment and two control treatments:

A: inoculation with *B. aryabhattai*;

V: coinoculation with *B. aryabhattai* + *A. brasilense*;

P: positive control (nitrogen fertilization without bioinputs);

B: negative control (without mineral N and without bioinputs).

All treatments involving bioinputs (A and V) received mineral fertilization according to the recommended management practices, while only the negative control treatment did not receive nitrogen fertilization.

Inoculants were applied immediately prior to sowing. For *B. aryabhattai*, strain CCT8087 was used (1 × 10^9^ CFU mL^−1^, 5 mL kg^−1^ of seeds), whereas coinoculation simultaneously included *A. brasilense* strains Ab-V5 and Ab-V6 (1 × 10^9^ CFU mL^−1^) associated with strain CCT8087 at the same application rate.

Each experimental plot occupied an area of 28.16 m^2^, and each subplot 7.04 m^2^, consisting of 42 plants arranged in seven rows. The six central plants were considered the useful sampling area.

The experimental design prioritized the evaluation of Bacillus-based inoculation, both alone and in combination with *Azospirillum brasilense*, to assess potential synergistic effects, while maintaining a feasible number of treatments under field conditions.

### 4.3. Experimental Management

The double-cross maize hybrid BRS2022, adapted to the semiarid climate conditions of Northeastern Brazil, was used in this study. Initial soil preparation consisted of disk harrowing to a depth of 0–30 cm. Fertilization followed the crop-specific recommendations [36], with the application of 30 kg ha^−1^ of N, 40 kg ha^−1^ of P_2_O_5_ and 20 kg ha^−1^ of K_2_O at sowing, in addition to 60 kg ha^−1^ of N and 20 kg ha^−1^ of K_2_O as topdressing. Urea, single superphosphate, and potassium chloride were used as nutrient sources.

Manual sowing was performed at a depth of 0.05 m, with a spacing of 0.50 m × 0.33 m, targeting a plant density of 60,600 plants ha^−1^. Thinning was carried out 10 days after emergence, maintaining one plant per hill. Weed control was performed by manual hoeing, and pests were managed preventively through the application of a sulfluramid-based ant bait.

**Figure 15:**
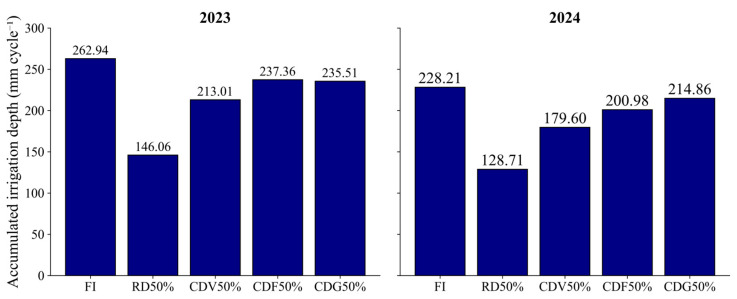
Cumulative irrigation depths applied under the different irrigation strategies evaluated during the first (2023) and second (2024) growing seasons. FI, full irrigation; RD50%, regular deficit; CDV50%, controlled deficit during the vegetative stage; CDF50%, controlled deficit during flowering and grain formation; CDG50%, controlled deficit during the grain-filling stage.

A localized drip irrigation system was used, consisting of 16 mm drip lines with emitters spaced at 0.30 m, a discharge rate of 1.6 L h^−1^ and an operating pressure of 10 m of water column. Irrigation water was obtained from a surface source with an electrical conductivity of approximately 0.75 dS m^−1^. Irrigation management was based on the Class A pan method, with estimation of reference evapotranspiration (ETo) and the use of crop coefficients (Kc) [37], adjusted according to phenological stage [38]. The cumulative irrigation depths applied under each treatment are shown in Figure 15.

When expressed in volumetric terms, in 2023, full irrigation (FI) corresponded to 2629.42 m^3^ ha^−1^, whereas RD50%, CDV50%, CDF50%, and CDG50% resulted in 1460.57, 2130.40, 2373.56, and 2355.09 m^3^ ha^−1^, respectively. In 2024, the applied volumes were: FI, 2282.12 m^3^ ha^−1^; RD50%, 1287.06 m^3^ ha^−1^; CDV50%, 1796.05 m^3^ ha^−1^; CDF50%, 2009.82 m^3^ ha^−1^; and CDG50%, 2148.62 m^3^ ha^−1^.

### 4.4. Variables Analyzed—Physiological Indices

Physiological evaluations included net photosynthetic rate (A, µmol m^−2^ s^−1^), transpiration rate (E, mol m^−2^ s^−1^), stomatal conductance (gs, mol m^−2^ s^−1^) and internal CO_2_ concentration (Ci, ppm), measured using a portable infrared gas analyzer (IRGA, LI-6400XT, LI-COR^®^, Lincoln, NE, USA). Measurements were taken on fully expanded leaves located in the middle third of the plant canopy between 9:00 and 11:00 a.m., at three time points during the first growing season (50, 64, and 79 days after sowing, DAS) and at two time points during the second cycle (55 and 72 DAS). The SPAD chlorophyll index was also determined using a SPAD-502 m (Minolta, Tokyo, Japan). Instantaneous carboxylation efficiency was estimated by the A/Ci ratio (µmol m^−2^ s^−1^/µmol mol^−1^).

### 4.5. Variables Analyzed—Yield Components and Water Use Efficiency

Harvest was performed at 100 and 104 days after sowing for the first (2023) and second (2024) growing seasons, respectively. The following variables were evaluated: fresh ear mass with husk (MFC, g), fresh ear mass without husk (MFS, g), ear diameter (ED, mm), ear length (EL, cm), number of kernel rows per ear (NR), number of kernels per row (NKR, kernels row^−1^), 100-kernel mass (M100, g), cob mass (CM, g), grain yield per plant (GYP, g plant^−1^) and grain yield (YIELD, kg ha^−1^).

The evaluation of ear mass both with and without husk was performed to distinguish between total ear biomass and the productive fraction more directly associated with grain yield.

For the estimation of grain yield per plant, kernels obtained after shelling were oven-dried in a forced-air circulation oven at 65 °C until constant mass. The material was then weighed on an analytical balance, and kernel mass was corrected to 13% moisture content.

Water use efficiency was determined through irrigation water productivity (IWP), calculated as the ratio between total grain yield (YIELD, kg ha^−1^) and the volume of irrigation water applied (m^3^ ha^−1^) for each treatment at the end of the crop cycle [39].

### 4.6. Variables Analyzed—Crop Sensitivity Coefficient to Water Deficit (Ky)

The crop sensitivity coefficient to water deficit (Ky) was calculated as the ratio between the relative reduction in grain yield and the relative reduction in evapotranspiration [40]. This parameter allows the estimation of crop response to water stress at different phenological stages.

### 4.7. Variables Analyzed—Economic-Social Analysis

For the economic analysis, gross production value (GPV), farmer income (FI), and the minimum area required to ensure social reproduction (MA-SR) were considered [41].

The evaluation of multiple agronomic, physiological, and economic variables was adopted to capture the complexity of plant responses to water deficit and bioinput application under semiarid conditions. This integrated approach allows for a more comprehensive assessment of crop performance beyond yield alone, including resource use efficiency and economic viability.

### 4.8. Statistical Analysis

Data were subjected to residual normality analysis using the Shapiro–Wilk test and homogeneity of variances using Bartlett’s test. Subsequently, analysis of variance (ANOVA) was performed using the F-test for each variable evaluated in each growing season. When significant, means were compared using Tukey’s test (*p* ≤ 0.05), employing R software version 4.1.2 (R Core Team, Vienna, Austria, 2024). Graphs were generated using SigmaPlot software (version 14.0).

## 5. Conclusions

This study demonstrated that the integration of controlled deficit irrigation strategies and the use of bioinputs can reconcile physiological, productive, and economic gains in maize cultivation under semiarid conditions.

The water deficit applied regularly throughout the crop cycle significantly reduced maize productivity. In contrast, controlled deficits imposed at specific growth stages, particularly during the vegetative stage (CDV50%), maintained yields close to those obtained under full irrigation (FI), while allowing greater irrigation water use efficiency (IWP).

Inoculation with *B. aryabhattai* and coinoculation with *B. aryabhattai* + *A. brasilense* promoted physiological adjustments associated with gas exchange and carboxylation efficiency. These effects were reflected in higher irrigation water productivity and, in specific production systems, increases in farmer income (FIc).

The results also indicated reduced crop sensitivity to water deficit (Ky) at less critical developmental stages, reinforcing the role of bioinputs as effective tools to mitigate the impacts of water stress.

From an economic perspective, the CDV50% strategy combined with coinoculation showed the best viability indicators, successfully reconciling grain yield, irrigation water productivity, and net farmer income. This combination appears particularly promising for family-based agricultural systems in semiarid regions.

Overall, the integration of bioinputs with controlled deficit irrigation represents a sustainable strategy with high potential for maize production in semiarid environments. Future research should expand evaluations across different environments, growing seasons, and maize genotypes, as well as incorporate environmental and socioeconomic indicators, in order to consolidate more robust technical recommendations for the agricultural sector.

From a practical perspective, the results highlight that the adoption of controlled deficit irrigation strategies, particularly when combined with bacterial inoculation, can contribute to more sustainable maize production systems in semiarid regions. These approaches allow for significant water savings while maintaining satisfactory yield levels, representing a viable alternative for optimizing resource use, especially under conditions of water scarcity. Such strategies are particularly relevant for smallholder farmers, as they enhance both water use efficiency and economic returns while promoting more resilient agricultural systems.

## Figures and Tables

**Table 1:** Summary of the analysis of variance based on mean squares for ear diameter (ED), ear length (EL), number of kernel rows per ear (NR), number of kernels per row (NKR), 100-kernel mass (M100), cob mass (CM), fresh ear mass with husk (MFC), fresh ear mass without husk (MFS), grain yield per plant (GYP), and grain yield (YIELD) of maize during the first (2023) and second (2024) growing seasons.

SV	ED	EL	NR	NKR	M100	CM	MFC	MFS	GYP	YIELD
First growing season—2023										
Blocks	5.75 ^ns^	5.44 ^ns^	2.01 ^ns^	20.35 ^ns^	16.81 ^ns^	16.76 ^ns^	3305.76 ^ns^	369.12 ^ns^	221.24 ^ns^	915,276.8 ^ns^
Ir	30.8 ^ns^	8.90 ^ns^	1.36 ^ns^	66.08 ^ns^	15.59 ^ns^	64.09 ^ns^	2445.13 ^ns^	2539.35 *	1571.82 *	6,656,914.1 *
Res. 1	10.24	4.47	1.35	26.59	8.94	22.89	3426.63	720.07	480.94	1,777,243.5
Bio	9.02 ^ns^	4.10 ^ns^	2.15 ^ns^	6.68 ^ns^	1.68 ^ns^	8.43 ^ns^	2563.68 ^ns^	106.08 ^ns^	387.02 ^ns^	298,959.2 ^ns^
Ir × Bio	4.79 ^ns^	5.26 ^ns^	2.45 ^ns^	20.09 ^ns^	5.67 ^ns^	9.81 ^ns^	3028.13 ^ns^	428.62 *	529.55 ^ns^	1,167,509.1 ^ns^
Res. 2	5.02	3.76	1.7	10.94	6.26	7.74	1868.28	210.56	331.96	599,408.7
Total	-	-	-	-	-	-	-	-	-	-
CV1 (%)	7.71	15.1	8.02	21.07	11.28	25.36	47.4	25.28	24.99	25.69
CV2 (%)	5.4	13.83	8.98	13.52	9.44	14.74	35	13.67	20.77	14.92
Second growing season—2024										
Blocks	13.98 *	5.60 ^ns^	6.68 *	60.81 *	13.69 ^ns^	40.16 ^ns^	4221.59 ^ns^	3618.58 *	1621.42 ^ns^	5,836,685.4 ^ns^
Ir	18.31 *	11.90 ^ns^	0.46 ^ns^	87.71 **	13.28 ^ns^	132.66 ^ns^	6914.56 *	5254.76 *	3028.71 *	10,902,086.5 *
Res. 1	3.4	4.30	1.55	15.20	9.15	44.8	1394.52	989.07	567.65	2,044,094.4
Bio	7.29 ^ns^	0.19 ^ns^	1.92 ^ns^	0.64 ^ns^	1.85 ^ns^	14.33 ^ns^	1205.72 ^ns^	582.82 ^ns^	290.20 ^ns^	1,044,288.7 ^ns^
Ir × Bio	2.98 ^ns^	1.35 ^ns^	1.32 ^ns^	5.66 ^ns^	2.31 ^ns^	16.56 ^ns^	810.03 ^ns^	479.98 ^ns^	189.86 ^ns^	683,564.3 ^ns^
Res. 2	5.14	1.66	2.13	10.81	6.55	23.77	901.19	605.62	366.17	1,210,387.9
Total	-	-	-	-	-	-	-	-	-	-
CV1 (%)	4.19	12.64	8.75	12.85	10.88	27.25	27.18	26.45	24.87	24.88
CV2 (%)	5.16	7.85	10.26	10.84	9.21	19.85	21.85	20.70	19.14	19.14

SV: Sources of variation; BIO: Bioinput; ** Significant by the F test at 1% probability; * Significant by the F test at 5% probability; ^ns^: not significant; CV: Coefficient of variation; Ir: Irrigation; Res.: Residual.

**Table 2:** Summary of the analysis of variance (mean squares) for A, E, gs, Ci, and A/Ci evaluated at three time points in the first growing season and two time points in the second growing season.

Variable		Mean Square			CV (%)
		Ir	Bio	Ir × Bio	
First growing season—2023					
A	50 DAS	65.62 ^ns^	37.13 ^ns^	26.38 ^ns^	21.37
	64 DAS	85.22 ^ns^	13.51 ^ns^	24.31 ^ns^	32.32
	79 DAS	13.596 ^ns^	2.387 ^ns^	23.145 ^ns^	15.03
E	50 DAS	2.745 ^ns^	0.386 ^ns^	0.305 ^ns^	27.61
	64 DAS	5.082 *	8.038 **	2.916 ^ns^	18.25
	79 DAS	1.512 ^ns^	0.274 ^ns^	0.599 ^ns^	21.56
gs	50 DAS	2.175 ^ns^	0.705 ^ns^	0.366 ^ns^	19.29
	64 DAS	0.2185 **	0.0896 **	0.0242 ^ns^	21.70
	79 DAS	18.451 ^ns^	0.5487 ^ns^	0.7657 ^ns^	12.18
Ci	50 DAS	435.5 ^ns^	748.5 ^ns^	382.7 ^ns^	11.11
	64 DAS	4363 **	788 ^ns^	2061 *	7.33
	79 DAS	10,638 ^ns^	841 ^ns^	1798 ^ns^	14.79
A/Ci	50 DAS	0.0006 ^ns^	0.0008 ^ns^	0.0003 ^ns^	22.71
	64 DAS	0.0013 ^ns^	0.0002 ^ns^	0.0011 ^ns^	25.83
	79 DAS	0.0003 ^ns^	0.0002 ^ns^	0.0007 ^ns^	26.54
Second growing season—2024					
A	55 DAS	44.17 ^ns^	57.21 ^ns^	61.13 ^ns^	25.93
	72 DAS	11.83 ^ns^	112.33 ^ns^	123.26 ^ns^	27.20
E	55 DAS	2.037 ^ns^	1.087 ^ns^	0.399 ^ns^	25.80
	72 DAS	4.192 ^ns^	2.036 ^ns^	2.442 ^ns^	22.47
gs	55 DAS	0.0095 ^ns^	0.0043 ^ns^	0.0024 ^ns^	22.38
	72 DAS	0.0151 ^ns^	0.0240 ^ns^	0.0150 ^ns^	20.65
Ci	55 DAS	1680 ^ns^	1823 ^ns^	3345 ^ns^	17.64
	72 DAS	1412 ^ns^	601 ^ns^	4431 *	18.96
A/Ci	55 DAS	0.146 ^ns^	0.165 ^ns^	0.1111 ^ns^	21.71
	72 DAS	0.0113 ^ns^	0.0114 ^ns^	0.0384 *	17.52

* Significant by the F test at 5% probability; ** Significant by the F test at 1% probability; ^ns^: not significant; CV: coefficient of variation; Ir: irrigation; Bio: bioinputs.

**Table 3:** Values of Yr, Ym, (1 − Yr/Ym), (1 − ETr/ETm), and the crop sensitivity coefficients (Ky) during the first and second growing seasons of maize.

First Growing Season—2023						
Treatments	Yr	Ym	(ETr/ETm)	[1 − (Yr/Ym)]	[1 − (ETr/ETm)]	Ky
RD50%-A	5266	5918	0.555	0.11	0.44	0.25
RD50%-V	4128	5761	0.555	0.28	0.44	0.64
RD50%-P	4352	6112	0.555	0.29	0.44	0.65
RD50%-B	4146	6385	0.555	0.35	0.44	0.79
CDV50%-A	4097	5918	0.810	0.31	0.19	1.62
CDV50%-V	5170	5761	0.810	0.10	0.19	0.54
CDV50%-P	5110	6112	0.810	0.16	0.19	0.86
CDV50%-B	4447	6385	0.810	0.30	0.19	1.60
CDF50%-A	5240	5918	0.903	0.11	0.10	1.18
CDF50%-P	6039	6112	0.903	0.01	0.10	0.12
CDF50%-B	4988	6385	0.903	0.22	0.10	2.25
CDG50%-A	4992	5918	0.896	0.16	0.10	1.50
CDG50%-V	5523	5761	0.896	0.04	0.10	0.40
CDG50%-P	4445	6112	0.896	0.27	0.10	2.61
CDG50%-B	5497	6385	0.896	0.14	0.10	1.33
Second Growing Season—2024						
Treatments	Yr	Ym	(ETr/ETm)	[1 − (Yr/Ym)]	[1 − (ETr/ETm)]	Ky
RD50%-A	5003	6808	0.558	0.27	0.44	0.60
RD50%-V	5242	6561	0.558	0.20	0.44	0.45
RD50%-P	6158	6284	0.558	0.02	0.44	0.05
RD50%-B	4830	6026	0.558	0.20	0.44	0.45
CDV50%-A	6106	6808	0.806	0.10	0.19	0.53
CDV50%-V	7026	6561	0.806	−0.07	0.19	−0.37
CDV50%-P	6106	6284	0.806	0.03	0.19	0.15
CDV50%-B	6626	6026	0.806	−0.10	0.19	−0.51
CDF50%-A	5360	6808	0.890	0.21	0.11	1.93
CDF50%-V	6512	6561	0.890	0.01	0.11	0.07
CDF50%-P	6030	6284	0.890	0.04	0.11	0.37
CDG50%-A	4257	6808	0.913	0.37	0.09	4.33
CDG50%-V	4839	6561	0.913	0.26	0.09	3.03
CDG50%-P	4518	6284	0.913	0.28	0.09	3.25
CDG50%-B	4390	6026	0.913	0.27	0.09	3.14

RD50%—Regular deficit throughout the entire crop cycle; CDV50%—Controlled deficit during the vegetative stage; CDF50%—Controlled deficit during the flowering and grain formation stage; CDG50%—Controlled deficit during the grain-filling stage; Ky—Crop sensitivity coefficient to water deficit (dimensionless); Yr—Actual crop yield under water deficit (kg ha^−1^); Ym—Maximum crop yield under full irrigation (kg ha^−1^); ETr—Actual crop evapotranspiration (mm); ETm—Maximum crop evapotranspiration under full irrigation (mm). Treatments CDF50%-V in 2023 and CDF50%-B in 2024 were excluded from the analysis because they presented negative values, which limit a physically meaningful interpretation of the results.

**Table 4:** Gross production value (GPV) and parameters used to calculate farmer income (FIc) for each production system in the maize growing season of 2023.

	Production Cycle 2023						
Treatments	Production (kg ha^−1^)	GPV	VA	ITR	J	S	FIc
FI-V	5761	6337.10	12,674.20	12.6	1001.92	633.6	11,026.08
FI-P	6112	6723.20	13,446.40	12.6	1001.92	633.6	11,798.28
FI-B	6385	7023.50	14,047.00	12.6	1001.92	633.6	12,398.88
FI-A	5918	6509.80	13,019.60	12.6	1001.92	633.6	11,371.48
RD50%-V	4128	4540.80	9081.60	12.6	1001.92	633.6	7433.48
RD50%-P	4352	4787.20	9574.40	12.6	1001.92	633.6	7926.28
RD50%-B	4146	4560.60	9121.20	12.6	1001.92	633.6	7473.08
RD50%-A	5266	5792.60	11,585.20	12.6	1001.92	633.6	9937.08
CDV50%-V	5170	5687.00	11,374.00	12.6	1001.92	633.6	9725.88
CDV50%-P	5110	5621.00	11,242.00	12.6	1001.92	633.6	9593.88
CDV50%-B	4447	4891.70	9783.40	12.6	1001.92	633.6	8135.28
CDV50%-A	4097	4506.70	9013.40	12.6	1001.92	633.6	7365.28
CDF50%-V	6193	6812.30	13,624.60	12.6	1001.92	633.6	11,976.48
CDF50%-P	6039	6642.90	13,285.80	12.6	1001.92	633.6	11,637.68
CDF50%-B	4988	5486.80	10,973.60	12.6	1001.92	633.6	9325.48
CDF50%-A	5240	5764.00	11,528.00	12.6	1001.92	633.6	9879.88
CDG50%-V	5523	6075.30	12,150.60	12.6	1001.92	633.6	10,502.48
CDG50%-P	4445	4889.50	9779.00	12.6	1001.92	633.6	8130.88
CDG50%-B	5497	6046.70	12,093.40	12.6	1001.92	633.6	10,445.28
CDG50%-A	4992	5491.20	10,982.40	12.6	1001.92	633.6	9334.28

FI, full irrigation; RD50%, regular deficit; CDV50%, controlled deficit during the vegetative stage; CDF50%, controlled deficit during flowering and grain formation; CDG50%, controlled deficit during the grain-filling stage. GPV, gross production value (R$); VA, value added (R$); ITR, rural land tax (R$); J, annual financing interest rate (R$); S, labor costs (R$); FIc, farmer income (R$).

**Table 5:** Gross production value (GPV) and parameters used to calculate farmer income (FIc) for each production system in the maize growing season of 2024.

	Production Cycle—2024						
Treatment	Production (kg ha^−1^)	GPV	VA	ITR	J	S	FIc
FI-A	6808	7488.80	14,977.60	12.6	1001.92	677.7	13,285.32
FI-V	6561	7217.10	14,434.20	12.6	1001.92	677.7	12,741.92
FI-P	6284	6912.40	13,824.80	12.6	1001.92	677.7	12,132.52
FI-B	6026	6628.60	13,257.20	12.6	1001.92	677.7	11,564.92
RD50%-A	5003	5503.30	11,006.60	12.6	1001.92	677.7	9314.32
RD50%-V	5242	5766.20	11,532.40	12.6	1001.92	677.7	9840.12
RD50%-P	6158	6773.80	13,547.60	12.6	1001.92	677.7	11,855.32
RD50%-B	4830	5313.00	10,626.00	12.6	1001.92	677.7	8933.72
CDV50%-A	6106	6716.60	13,433.20	12.6	1001.92	677.7	11,740.92
CDV50%-V	7026	7728.60	15,457.20	12.6	1001.92	677.7	13,764.92
CDV50%-P	6106	6716.60	13,433.20	12.6	1001.92	677.7	11,740.92
CDV50%-B	6626	7288.60	14,577.20	12.6	1001.92	677.7	12,884.92
CDF50%-A	5360	5896.00	11,792.00	12.6	1001.92	677.7	10,099.72
CDF50%-V	6512	7163.20	14,326.40	12.6	1001.92	677.7	12,634.12
CDF50%-P	6030	6633.00	13,266.00	12.6	1001.92	677.7	11,573.72
CDF50%-B	6165	6781.50	13,563.00	12.6	1001.92	677.7	11,870.72
CDG50%-A	4257	4682.70	9365.40	12.6	1001.92	677.7	7673.12
CDG50%-V	4839	5322.90	10,645.80	12.6	1001.92	677.7	8953.52
CDG50%-P	4518	4969.80	9939.60	12.6	1001.92	677.7	8247.32
CDG50%-B	4390	4829.00	9658.00	12.6	1001.92	677.7	7965.72

FI, full irrigation; RD50%, regular deficit; CDV50%, controlled deficit during the vegetative stage; CDF50%, controlled deficit during flowering and grain formation; CDG50%, controlled deficit during the grain-filling stage. GPV, gross production value (R$); VA, value added (R$); ITR, rural land tax (R$); J, annual financing interest rate (R$); S, labor costs (R$); FIc, farmer income (R$).

**Table 6:** Linear equations relating farmer income (FIc) to cultivated area.

Treatment	Equations—2023	Equations—2024
FI-A	y = 11,026.08x	y = 13,285.32x
FI-V	y = 11,798.28x	y = 12,741.92x
FI-P	y = 12,398.88x	y = 12,132.52x
FI-B	y = 11,371.48x	y = 11,564.92x
RD50%-A	y = 7433.48x	y = 9314.32x
RD50%-V	y = 7926.28x	y = 9840.12x
RD50%-P	y = 7473.08x	y = 11,855.32x
RD50%-B	y = 9937.08x	y = 8933.72x
CDV50%-A	y = 9725.88x	y = 11,740.92x
CDV50%-V	y = 9593.88x	y = 13,764.92x
CDV50%-P	y = 8135.28x	y = 11,740.92x
CDV50%-B	y = 7365.28x	y = 12,884.92x
CDF50%-A	y = 11,976.48x	y = 10,099.72x
CDF50%-V	y = 11,637.68x	y = 12,634.12x
CDF50%-P	y = 9325.48x	y = 11,573.72x
CDF50%-B	y = 9879.88x	y = 11,870.72x
CDG50%-A	y = 10,502.48x	y = 7673.12x
CDG50%-V	y = 8130.88x	y = 8953.52x
CDG50%-P	y = 10,445.28x	y = 8247.32x
CDG50%-B	y = 9334.28x	y = 7965.72x

FI, full irrigation; RD50%, regular deficit; CDV50%, controlled deficit during the vegetative stage; CDF50%, controlled deficit during flowering and grain formation; CDG50%, controlled deficit during the grain-filling stage. y, farmer income (FIc, R$); x, cultivated area (ha).

**Table 7:** Minimum area required to ensure social reproduction (MA-SR) under different treatments in 2023 and 2024.

Treatment	MA-SR—2023 (ha)	MA-SR—2024 (ha)
FI-A	7.09	6.86
FI-V	6.62	7.15
FI-P	6.30	7.51
FI-B	6.87	7.88
RD50%-A	10.51	9.78
RD50%-V	9.86	9.26
RD50%-P	10.45	7.68
RD50%-B	7.86	10.20
CDV50%-A	8.03	7.76
CDV50%-V	8.14	6.62
CDV50%-P	9.60	7.76
CDV50%-B	10.61	7.07
CDF50%-A	6.52	9.02
CDF50%-V	6.71	7.21
CDF50%-P	8.38	7.87
CDF50%-B	7.91	7.67
CDG50%-A	7.44	11.87
CDG50%-V	9.61	10.17
CDG50%-P	7.48	11.04
CDG50%-B	8.37	11.43

FI, full irrigation; RD50%, regular deficit; CDV50%, controlled deficit during the vegetative stage; CDF50%, controlled deficit during flowering and grain formation; CDG50%, controlled deficit during the grain-filling stage. MA-SR, minimum area required to ensure social reproduction (ha).

**Table 8:** Physical and chemical characterization of the soil in the experimental area at the 0–20 cm layer prior to field crop establishment.

Attribute	Value	Attribute	Value
Sand (%)	37.1	Base saturation, V (%)	90
Silt (%)	43.2	Organic matter (g kg^−1^)	16.45
Clay (%)	19.7	Available P (mg kg^−1^)	37
Natural clay (g kg^−1^)	160	Ca^2+^ cmol_c_ kg^−1^	11.2
Textural class	Loam	Mg^2+^ cmol_c_ kg^−1^	1.6
Soil bulk density (g cm^−3^)	1.25	Na^+^ cmol_c_ kg^−1^	0.31
pH	7	K^+^ cmol_c_ kg^−1^	0.57
ECse (dS m^−1^)	0.38	H^+^ + Al^3+^ cmol_c_ kg^−1^	1.49
C (g kg^−1^)	9.54	Al^3+^ cmol_c_ kg^−1^	0.1
N (g kg^−1^)	1.05	Sum of bases, S cmol_c_ kg^−1^	13.7
Exchangeable sodium percentage, ESP (%)	2	CEC at pH 7.0 cmol_c_ kg^−1^	15.2

## Data Availability

The original contributions presented in this study are included in the article/Supplementary Materials. Further inquiries can be directed to the corresponding author (agrodanilonogueira@gmail.com).

## Supplementary Materials

The following supporting information can be downloaded at: https://www.mdpi.com/article/10.3390/plants15091309/s1.
